# Performance Enhancement of Drone Acoustic Source Localization Through Distributed Microphone Arrays

**DOI:** 10.3390/s25061928

**Published:** 2025-03-20

**Authors:** Jaejun Lim, Jaehan Joo, Suk Chan Kim

**Affiliations:** Department of Electrical and Electronic Engineering, Pusan National University, Busan 46241, Republic of Korea; limlim258@pusan.ac.kr (J.L.); jhjoo2018@pusan.ac.kr (J.J.)

**Keywords:** drone localization, anti-drone system, microphone array, generalized cross-correlation phase transform

## Abstract

This paper presents a novel localization method that leverages two sets of distributed microphone arrays using the Generalized Cross-Correlation Phase Transform (GCC-PHAT) technique to improve the performance of anti-drone systems. In contrast to conventional sound source localization techniques, the proposed approach enhances localization accuracy by precisely estimating the azimuth angle while considering the unique acoustic characteristics of drones. The effectiveness of the proposed method was validated through both simulations and field tests. Simulation results revealed that, in ideal channel conditions, the proposed method significantly reduced the mean and variance of localization errors compared to existing techniques, resulting in more accurate positioning. Furthermore, in noisy environments, the proposed approach consistently outperformed the comparison method across various Signal-to-Noise Ratio (SNR) levels, achieving up to 2.13 m of improvement at SNR levels above 0 dB. While the comparison method exhibited decreased localization accuracy along the y-axis and z-axis, the proposed method maintained stable performance across all axes by effectively distinguishing between azimuth and elevation angles. Field test results closely mirrored the simulation outcomes, further confirming the robustness and reliability of the proposed localization approach.

## 1. Introduction

In recent years, the field of drone technology has experienced remarkable advancements, drawing substantial attention from various industries. Due to their compact size and high maneuverability, drones have found widespread applications in areas such as aerial photography, logistics, communications, and environmental monitoring [1,2,3]. Although these features enable numerous positive applications, they also raise concerns about potential misuse, with increasing reports of drones being used for malicious purposes. Incidents involving unauthorized drone operations near airports and attacks on public institutions or individuals have been documented [4,5]. Consequently, the development of effective anti-drone systems has become critical, both in civilian and military contexts, to prevent accidents and enable rapid response to such threats.

An anti-drone system functions by detecting unauthorized drones and tracking their locations to prevent malicious activities. Accurate localization of a drone is a key component in developing an effective anti-drone system, and numerous signal processing and localization techniques have been actively explored for this purpose. Prominent technologies include ultraviolet (UV) detection, thermal imaging, magnetic sensors, and acoustic source-based localization methods. UV detection is effective in identifying drones by detecting UV signatures, especially in low-light conditions. Thermal imaging is beneficial for detecting drones based on heat emissions, making it suitable for night-time operations or situations with visual obstructions. Magnetic sensors are used to detect the metallic components of drones, which is advantageous in environments where visual and acoustic signals are compromised. Acoustic source-based localization methods are based on the unique sounds produced by drone propellers, providing a resilient approach in dynamic environments with frequent visual obstructions. Each technique presents distinct advantages and limitations, making them suitable for different environments and operating conditions [4,5,6]. Among these, acoustic source-based localization methods are particularly effective for drone detection as they are relatively resilient to environmental changes and provide high localization accuracy. Although acoustic signals may overlap with ambient noise, potentially leading to false detections, the characteristic sounds produced by drone engines or propellers offer a reliable means of tracking the sound source with precision. Specific filtering techniques, such as bandpass filtering, are typically employed to differentiate drone sounds from background noise, enhancing detection reliability. However, advanced methods such as spectral subtraction and adaptive noise cancellation were not specifically considered in this study and are potential areas for future exploration.

Acoustic source localization is traditionally performed using triangulation methods based on Time of Arrival (TOA), time difference of arrival (TDOA), and angle of arrival (AOA) measurements. These techniques are widely used in various localization applications due to their established high accuracy and robustness [7,8,9,10,11,12]. Among these approaches, GCC-PHAT, as described by Pourmohammad and Ahadi, coupled with array signal processing, enables efficient and precise localization [12]. They proposed a real-time, high-accuracy 3D sound source localization technique using a simple four-microphone arrangement, which demonstrated the feasibility of achieving rapid localization with minimal hardware complexity. However, their method estimates AOA as azimuth, leading to a degree of inaccuracy because this simplification overlooks the intricate spatial variations necessary for accurately distinguishing three-dimensional positional attributes, especially in scenarios involving rapidly moving targets such as drones [12]. For instance, when a drone rapidly changes altitude, the incorrect estimation of azimuth can lead to significant localization errors, making it difficult to accurately track the drone’s real-time position and potentially causing delays in response actions. Calculating AOA as azimuth results in angular errors since the AOA does not accurately represent the true azimuth angle. In conventional acoustic source localization, the difference between AOA and azimuth is relatively small and may not cause significant performance deterioration. However, in the case of drones, where the sound source is at a very high altitude, the discrepancy between AOA and azimuth becomes more pronounced, causing localization errors. Therefore, it is crucial to perform localization while considering these differences.

Array signal processing is critical for improving localization accuracy and is applied in numerous advanced research fields [13,14,15,16]. Although azimuth can be effectively estimated using array signal processing, determining both AOA and azimuth separately with a single set of microphones leads to increased signal processing complexity and higher error rates. To address these challenges, we employ two sets of microphone arrays to achieve both computational simplicity and enhanced accuracy.

The two sets of microphone arrays were positioned symmetrically on either side of the origin, each at a distance of 2 m. This distance was chosen to optimize the balance between spatial separation for effective triangulation and simplifying computational overhead, thereby enhancing localization performance in high-altitude scenarios. By adopting this configuration, we were able to develop a robust and efficient localization technique that effectively mitigates the computational burden while maintaining high accuracy, particularly for high-altitude drone localization.

In this study, we propose a localization system that estimates the position of a drone by calculating both azimuth and elevation angles using two distributed microphone arrays, combined with TDOA measurements obtained through the GCC-PHAT technique. Unlike existing methods that often estimate AOA as azimuth or rely on single-array setups, our approach uses dual arrays to achieve higher accuracy and reduce computational complexity. This configuration mitigates angular errors inherent in traditional approaches for tracking drones at high altitudes. The effectiveness of the proposed method is validated through a series of simulations and experimental evaluations.

## 2. Related Work

Sound source localization (SSL) has been widely studied in the literature, with TDOA and Generalized Cross-Correlation Phase Transform (GCC-PHAT) being the most commonly employed techniques [1,2]. These methods have been extensively utilized in various applications, including speech recognition, surveillance, and, more recently, drone detection.

One of the most notable studies in this field is the work by Ali Pourmohammad et al. [12], who proposed a real-time high-accuracy 3D localization system based on a four-microphone array. Their method estimates the azimuth angle of a sound source using TDOA measurements, achieving reasonable localization accuracy in controlled environments. However, a major limitation of this approach is its inability to effectively capture elevation information, which is critical for applications involving airborne targets such as drones.

Several studies have attempted to address these limitations by improving microphone array configurations. Lee and Park [17] introduced a phased microphone array mounted on a drone to enhance acoustic source localization. Their work demonstrated increased detection accuracy, particularly for sources positioned at varying altitudes. Similarly, Kim and Choi [18] investigated a multi-microphone array system onboard UAVs, showing improvements in real-time tracking of nearby drones using advanced array signal processing techniques.

Beyond microphone configurations, recent research has also explored sensor fusion and machine learning techniques to further enhance localization accuracy. Wang and Chen [19] proposed a drone detection system combining fiber-optic acoustic sensors with distributed microphone arrays, significantly improving robustness against background noise. Meanwhile, Smith and Brown [20] applied deep learning-based 3D localization in urban environments, demonstrating that neural networks can effectively compensate for multi-path interference and noise.

Despite these advancements, accurate 3D localization in large-scale, real-world environments remains a challenge. Existing approaches either require a large number of microphones, which increases hardware complexity, or struggle with calibration issues in distributed setups. This highlights the need for a robust and cost-efficient localization method that can accurately estimate both azimuth and elevation angles.

### 2.1. Motivation and Contribution

In drone detection systems that require wide-area coverage, multiple microphone arrays must be deployed to enhance detection capabilities. The presence of multiple arrays enables performance improvements through inter-array calibration, while simultaneously ensuring that reducing the number of microphones per array remains economically advantageous.

**Figure 1:**
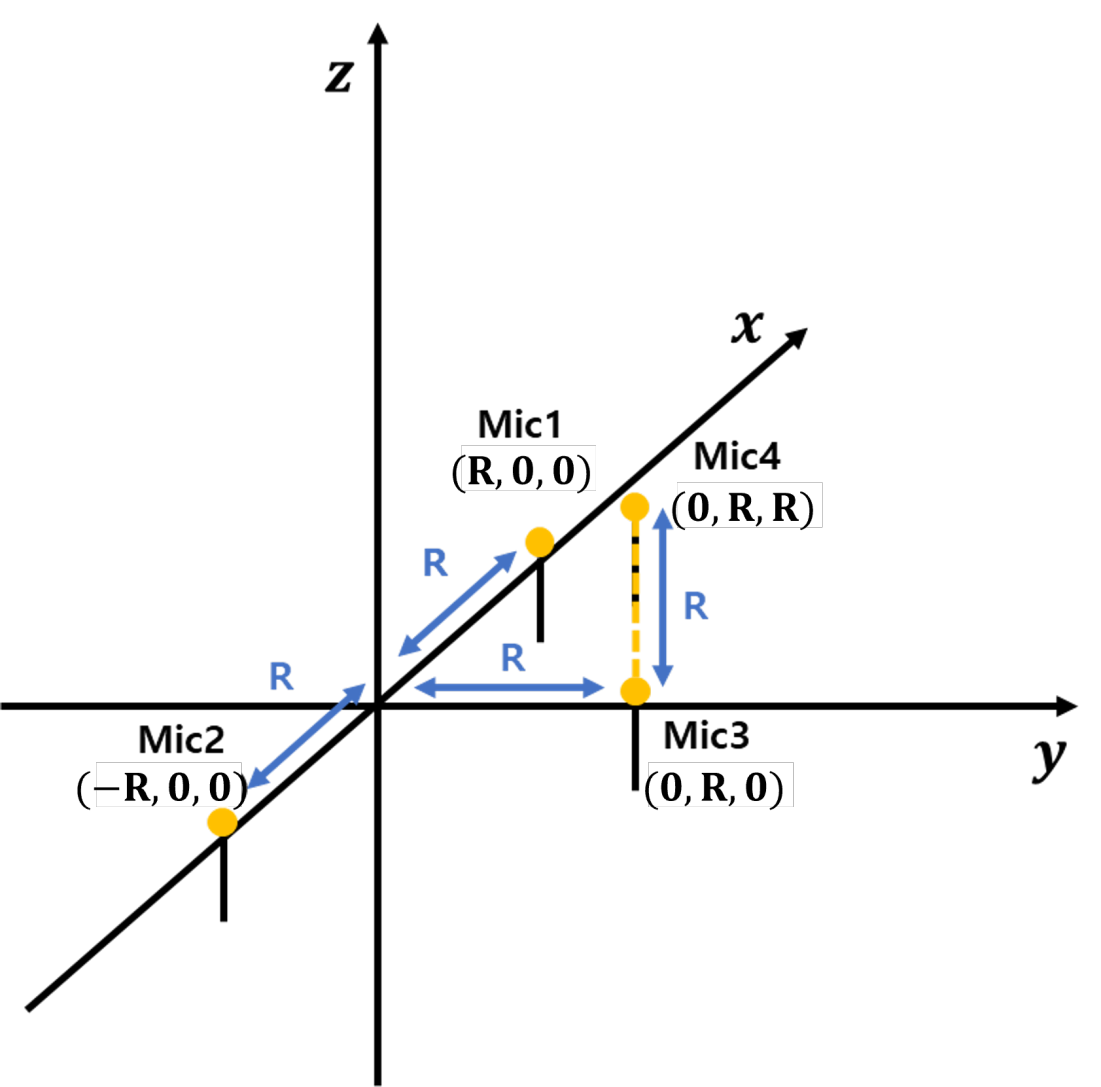
The microphone array configuration utilized for three-dimensional localization of the drone. The array consists of four microphones strategically positioned to facilitate accurate estimation of both the AOA and elevation. Specifically, microphones 1 and 2 are paired to calculate the AOA, while microphones 3 and 4 are used to determine the elevation. This configuration enhances the overall precision of the localization algorithm by providing robust spatial data from multiple reference points.

Based on these considerations, we selected the Ali method as a reference due to its low-complexity implementation and real-time processing capabilities. However, as mentioned earlier, the Ali method suffers from significant localization errors for high-altitude sound sources, such as drones. The microphone array used in this study is shown in Figure 1. These errors can result in substantial inaccuracies in drone tracking and pose challenges in post-processing corrections.

To address these issues, we propose a novel approach that utilizes two distributed microphone arrays to enhance 3D localization accuracy. This method not only improves the localization performance of distributed sensor networks but also contributes to reducing hardware costs in large-scale detection systems.

Our key contributions are summarized as follows:

- An extension of the GCC-PHAT-based TDOA framework to accurately compute both azimuth and elevation angles.
- The development of a low-complexity hyperbolic intersection algorithm for real-time 3D localization.
- Performance evaluation comparing our method with existing approaches, demonstrating improved accuracy and robustness in noisy environments.
- The enhancement of distributed sensor networks, enabling more efficient deployment of microphone arrays for large-scale drone detection.

### 2.2. GCC-PHAT-Based TDOA Estimation

Acoustic source localization techniques primarily utilize GCC-PHAT with TDOA for high-precision positioning. These methods rely on correlation functions to assess the dependency between signal pairs, such as identifying time delays or phase relationships. Correlation quantifies the similarity between stochastic processes, revealing temporal or spectral dependencies within signals. It is categorized into auto-correlation and cross-correlation, depending on whether the reference signal matches the target signal.

The auto-correlation function measures a signal’s similarity to its time-shifted version, aiding in the detection of periodic or repetitive patterns. In contrast, cross-correlation evaluates the similarity between two distinct signals, enabling the analysis of their relationship in the time or frequency domain. This helps identify time delays and synchronization across signals. The mathematical formulation of the auto-correlation function in the time domain is given in Equation (1).

(1) $$R_{xx}\left(\tau \right)=\int_{-\infty }^{\infty }x\left(t\right)x\left(t+\tau \right)dt.$$

In this case, x(t) denotes the received signal, $R_{xx}\left(\tau \right)$ represents its auto-correlation function, and τ corresponds to the time delay. By leveraging the duality between time and frequency domains, $R_{xx}\left(\tau \right)$ can also be expressed in the frequency domain, as shown in Equation (2).

(2) $$R_{xx}\left(\tau \right)=\int_{-\infty }^{\infty }X\left(f\right)X{\left(f\right)}^{*}e^{j2\pi f}df.$$

Here, X(f) denotes the Fourier transform of x(t). Additionally, the cross-correlation function $R_{xy}\left(\tau \right)$ between two signals x(t) and y(t) is defined as in Equation (3).

(3) $$R_{xy}\left(\tau \right)=\int_{-\infty }^{\infty }x\left(t\right)y\left(t+\tau \right)dt$$

If y(t) is considered as the delayed version of x(t), the cross-correlation function reaches its maximum at the point where the time delay matches. This is similar to the concept where the auto-correlation function reaches its maximum when there is no time delay. Therefore, the time delay difference between x(t) and y(t) can be represented by Equation (4).

(4) $$\tau_{12}=\arg\max_{\tau }R_{12}\left(\tau \right)$$

By applying the cross-correlation method, the TDOA between microphones can be accurately measured. The TDOA is essential in drone localization, as it represents the relative propagation time differences of signals received at each microphone. These data are critical for accurately determining the drone’s position in three-dimensional space. However, for low-frequency signals, such as those emitted by acoustic sources, the performance of the conventional cross-correlation approach tends to deteriorate [21]. This occurs because low-frequency signals have longer wavelengths, reducing spatial resolution and making it harder to distinguish between closely spaced sound sources, leading to increased interference and decreased accuracy. The long wavelengths associated with low-frequency signals also increase the likelihood of interference and distortion within the microphone array. These challenges are further exacerbated in environments with significant noise or multi-path propagation, where signal reflections and noise can heavily impact the accuracy of TDOA estimation.

To improve the accuracy of TDOA estimation, it is crucial to mitigate the influence of low-frequency components. The GCC-PHAT method is widely adopted to address this challenge. This approach determines time delay by utilizing the phase information of the signal in the frequency domain. By focusing on phase rather than amplitude, the method reduces the impact of low-frequency interference, enabling more accurate time delay estimation.

The application of the PHAT weighting function further refines this process by eliminating amplitude information, which enhances robustness against noise, and calculating the correlation solely based on phase. This technique ensures reliable delay estimation even in the presence of noise and multi-path effects. When the angular frequency is defined as ω=2πf, the GCC-PHAT function is mathematically expressed as shown in Equation (5).

(5) $$R_{xy}^{PHAT}\left(\tau \right)=\frac{1}{2\pi }\int_{-\infty }^{\infty }H_{PHAT}\left(f\right)X\left(\omega \right)Y{\left(\omega \right)}^{*}dt$$

Here, $H_{PHAT}\left(f\right)$ is the PHAT weighting function, which is used to remove the amplitude component and can be expressed as in Equation (6).

(6) $$H_{PHAT}\left(f\right)=\frac{1}{|X\left(f\right)Y^{*}\left(f\right)|}$$

Furthermore, by utilizing the duality between the time and frequency domains, it can be expressed as Equation (7).

(7) $$R_{xy}^{PHAT}\left(\tau \right)=F^{-1}\left\{\frac{X\left(f\right)Y^{*}\left(f\right)}{|X\left(f\right)Y^{*}\left(f\right)|}\right\}$$

This method simplifies the complex convolution operations involved in signal processing, enabling fast and accurate TDOA estimation. In this study, TDOA estimation was conducted using the GCC-PHAT technique. A system was designed and implemented to capture and analyze drone acoustic signals in an outdoor environment, employing GCC-PHAT alongside distributed microphone arrays.

### 2.3. Angle of Arrival Estimation and Hyperbolic Intersection-Based Localization

In this study, the TDOA is estimated between microphones using the GCC-PHAT method, which subsequently enables the calculation of the AOA. The AOA is given by

(8) $$\cos\left(\phi \right)=\frac{d_{2}-d_{1}}{D}=\frac{\left(t_{2}-t_{1}\right)v_{\mathrm{sound}}}{D}=\frac{\tau_{21}v_{\mathrm{sound}}}{D}.$$

where $v_{\mathrm{sound}}$ denotes the speed of sound in air and $\tau_{21}$ represents the time delay obtained from the cross-correlation of two received signals. However, determining the exact source position requires additional information. The distance between the source and each microphone can be expressed as:

(9) $$d_{1}=\sqrt{{\left(x_{s}-x_{1}\right)}^{2}+{\left(y_{s}-y_{1}\right)}^{2}}$$

(10) $$d_{2}=\sqrt{{\left(x_{s}-x_{2}\right)}^{2}+{\left(y_{s}-y_{2}\right)}^{2}}$$

where $x_{s}$ and $y_{s}$ define the drone’s coordinates. The difference in these distances is given by:

(11) $$d_{2}-d_{1}=\tau_{21}v_{\mathrm{sound}}.$$

Rewriting in terms of *x* and *y* instead of $x_{s}$ and $y_{s}$, we obtain:

(12) $$\tau_{21}=\frac{\sqrt{{\left(x-x_{2}\right)}^{2}+{\left(y-y_{2}\right)}^{2}}-\sqrt{{\left(x-x_{1}\right)}^{2}+{\left(y-y_{1}\right)}^{2}}}{v_{\mathrm{sound}}}.$$

Since this equation contains two unknowns, *x* and *y*, additional constraints are required. Assuming both microphones are equidistant from the origin, with a separation distance D=2R along the x-axis, the equation simplifies to:

(13) $$\tau_{21}=\frac{\sqrt{{\left(x+R\right)}^{2}+y^{2}}-\sqrt{{\left(x-R\right)}^{2}+y^{2}}}{v_{\mathrm{sound}}}.$$

**Figure 2:**
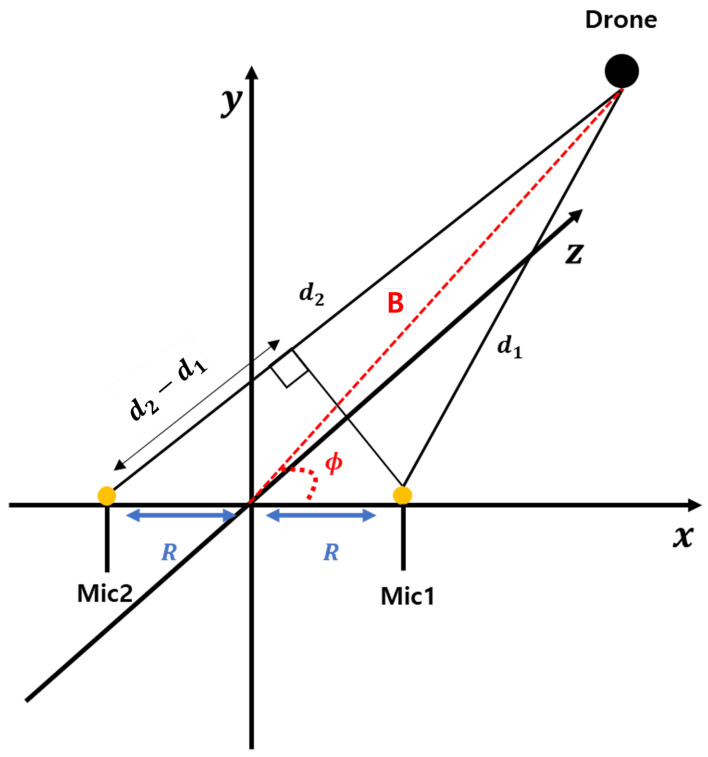
AOA approximation in a far-field scenario. This figure illustrates the process of estimating the AOA under far-field conditions. The approach assumes that incoming sound waves at the microphone array are nearly parallel, allowing for precise AOA determination by analyzing the time delay between received signals.

Rearranging and simplifying the equation leads to:

(14) $$\left\{\begin{array}{c} y^{2}=ax^{2}+b\\ a=\frac{4R^{2}}{v_{\mathrm{sound}}^{2}\tau_{21}^{2}}-1\\ b=\frac{v_{\mathrm{sound}}^{2}\tau_{21}^{2}}{4}-R^{2} \end{array}\right.$$

where *y* follows a hyperbolic geometric distribution relative to *x*, as depicted in Figure 2.

To determine the exact coordinates *x* and *y*, a second equation is needed by incorporating a third microphone:

(15) $$\left\{\begin{array}{c} \tau_{21}=\frac{\sqrt{{\left(x-x_{2}\right)}^{2}+{\left(y-y_{2}\right)}^{2}}-\sqrt{{\left(x-x_{1}\right)}^{2}+{\left(y-y_{1}\right)}^{2}}}{v_{\mathrm{sound}}}\;\\ \tau_{31}=\frac{\sqrt{{\left(x-x_{3}\right)}^{2}+{\left(y-y_{3}\right)}^{2}}-\sqrt{{\left(x-x_{1}\right)}^{2}+{\left(y-y_{1}\right)}^{2}}}{v_{\mathrm{sound}}}. \end{array}\right.$$

Since these are nonlinear equations, solving them requires a hyperbolic intersection approach, which typically involves numerical methods. Consequently, this increases computational complexity in localization, and there is a possibility that the solution may not converge.

### 2.4. Simplified Localization Calculations

**Figure 3:**
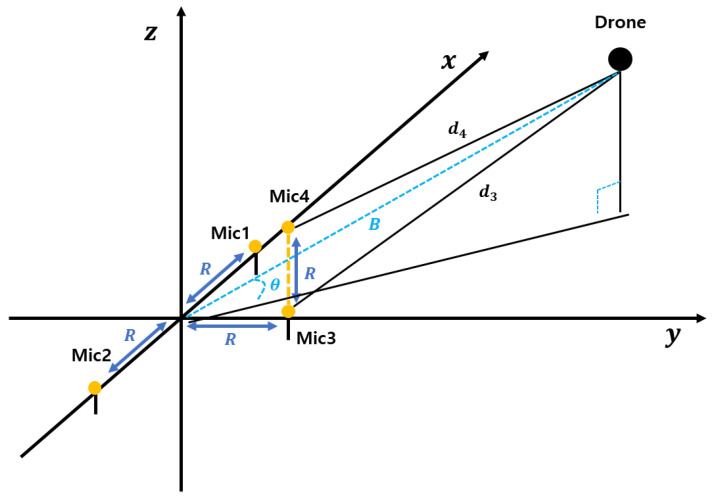
Elevation angle approximation process in a far-field environment. Similar to the process in Figure 1, this figure demonstrates the methodology for approximating the elevation angle under far-field conditions. The sound waves are assumed to be nearly parallel when reaching the microphone array, enabling the estimation of the elevation angle based on the time delay between signals captured by vertically aligned microphones. This configuration allows for precise calculation of the elevation in three-dimensional space.

Due to the complexity of solving Equation (15), we adopt a more practical microphone placement configuration, as illustrated in Figure 3. Based on this arrangement, we redefine the distance differences, leading to the following time delay expressions between microphone pairs:

(16) $$\tau_{12}=\frac{\sqrt{{\left(x-x_{1}\right)}^{2}+{\left(y-y_{1}\right)}^{2}}-\sqrt{{\left(x-x_{2}\right)}^{2}+{\left(y-y_{2}\right)}^{2}}}{v_{\mathrm{sound}}}$$

(17) $$\tau_{13}=\frac{\sqrt{{\left(x-x_{1}\right)}^{2}+{\left(y-y_{1}\right)}^{2}}-\sqrt{{\left(x-x_{3}\right)}^{2}+{\left(y-y_{3}\right)}^{2}}}{v_{\mathrm{sound}}}$$

(18) $$\tau_{23}=\frac{\sqrt{{\left(x-x_{2}\right)}^{2}+{\left(y-y_{2}\right)}^{2}}-\sqrt{{\left(x-x_{3}\right)}^{2}+{\left(y-y_{3}\right)}^{2}}}{v_{\mathrm{sound}}}$$

Given Equations (17) and (18) with two unknowns, *x* and *y*, we can simplify the problem by leveraging the fact that for each of the three sources, either *x* or *y* is zero. This allows us to rewrite Equations (17) and (18) as:

(19) $$\left\{\begin{array}{c} \tau_{13}=\frac{\sqrt{{\left(x-R\right)}^{2}+y^{2}}-\sqrt{x^{2}+{\left(y-R\right)}^{2}}}{v_{\mathrm{sound}}}\;\\ \tau_{23}=\frac{\sqrt{{\left(x-R\right)}^{2}+y^{2}}-\sqrt{x^{2}+{\left(y-R\right)}^{2}}}{v_{\mathrm{sound}}} \end{array}\right.$$

(20) $$\Rightarrow \left\{\begin{array}{c} \sqrt{x^{2}+{\left(y-R\right)}^{2}}=\frac{R}{v_{\mathrm{sound}}\tau_{13}}\left(y-x\right)-\frac{v_{\mathrm{sound}}\tau_{13}}{2}\;\\ \sqrt{x^{2}+{\left(y-R\right)}^{2}}=\frac{R}{v_{\mathrm{sound}}\tau_{23}}\left(y+x\right)-\frac{v_{\mathrm{sound}}\tau_{23}}{2} \end{array}\right.$$

(21) $$\Rightarrow \left\{\begin{array}{c} y=a_{1}x+b_{1}\\ a_{1}=\frac{\tau_{23}+\tau_{13}}{\tau_{23}-\tau_{13}}\;\\ b_{1}=-\frac{v_{\mathrm{sound}}^{2}}{2R}\left(\tau_{23}\tau_{13}\right). \end{array}\right.$$

Alternatively, solving Equations (16) and (17) with the same constraint (one coordinate being zero) allows us to rewrite them as:

(22) $$\left\{\begin{array}{c} \tau_{12}=\frac{\sqrt{{\left(x-R\right)}^{2}+y^{2}}-\sqrt{{\left(x+R\right)}^{2}+y^{2}}}{v_{\mathrm{sound}}}\;\\ \tau_{13}=\frac{\sqrt{{\left(x-R\right)}^{2}+y^{2}}-\sqrt{x^{2}+{\left(y-R\right)}^{2}}}{v_{\mathrm{sound}}} \end{array}\right.$$

(23) $$\Rightarrow \left\{\begin{array}{c} \sqrt{{\left(x-R\right)}^{2}+y^{2}}=\frac{-2R}{v_{\mathrm{sound}}\tau_{12}}x+\frac{v_{\mathrm{sound}}\tau_{12}}{2}\;\\ \sqrt{{\left(x-R\right)}^{2}+y^{2}}=\frac{R}{v_{\mathrm{sound}}\tau_{13}}\left(y-x\right)+\frac{v_{\mathrm{sound}}\tau_{13}}{2} \end{array}\right.$$

(24) $$\Rightarrow \left\{\begin{array}{c} y=a_{2}x+b_{2}\\ a_{2}=1-\frac{2\tau_{13}}{\tau_{12}}\;\\ b_{2}=\frac{v_{\mathrm{sound}}^{2}}{2R}\left(\tau_{12}-\tau_{13}\right)\tau_{13}. \end{array}\right.$$

Similarly, solving Equations (16) and (18) under the same assumption yields:

(25) $$\left\{\begin{array}{c} \tau_{12}=\frac{\sqrt{{\left(x-R\right)}^{2}+y^{2}}-\sqrt{{\left(x+R\right)}^{2}+y^{2}}}{v_{\mathrm{sound}}}\;\\ \tau_{23}=\frac{\sqrt{{\left(x+R\right)}^{2}+y^{2}}-\sqrt{x^{2}+{\left(y-R\right)}^{2}}}{v_{\mathrm{sound}}} \end{array}\right.$$

(26) $$\Rightarrow \left\{\begin{array}{c} \sqrt{{\left(x+R\right)}^{2}+y^{2}}=\frac{-2R}{v_{\mathrm{sound}}\tau_{12}}x-\frac{v_{\mathrm{sound}}\tau_{12}}{2}\;\\ \sqrt{{\left(x+R\right)}^{2}+y^{2}}=\frac{R}{v_{\mathrm{sound}}\tau_{23}}\left(y+x\right)+\frac{v_{\mathrm{sound}}\tau_{23}}{2} \end{array}\right.$$

(27) $$\Rightarrow \left\{\begin{array}{c} y=a_{3}x+b_{3}\;\\ a_{3}=\left(1+\frac{2\tau_{23}}{\tau_{12}}\right)\;\\ b_{3}=\frac{v_{\mathrm{sound}}^{2}}{2R}\left(\tau_{12}+\tau_{23}\right)\tau_{23}. \end{array}\right.$$

By replacing *a* with $a_{1},a_{2},$ or $a_{3}$ and similarly *b* with $b_{1},b_{2},$ or $b_{3}$, the source position is constrained to the line y=ax+b, as illustrated in Figure 3. This allows us to employ four different approaches to determine the source location:

1. Consider the incident line y=a·x+b and the line passing through the origin (line B in Figure 2; see Figure 3). The time delay between the second and first microphone signals, $(s_{2}\left(t\right)$ and $s_{1}\left(t\right))$, is denoted as $\tau_{12}$. Given that the angle of arrival is ϕ, we can determine it at the origin using the following equation:
  (28) $$\Phi =90^{\circ }-\cos^{-1}\left(\sqrt{1-{\left(\frac{d_{1}-d_{2}}{2R}\right)}^{2}}\right)$$
  (29) $$\Rightarrow \Phi =90^{\circ }-\cos^{-1}\left(\sqrt{1-{\left(\frac{v_{\mathrm{sound}}\tau_{12}}{2R}\right)}^{2}}\right)$$
  Assuming the source is at a distance *r* from the origin, its coordinates can be expressed as:
  (30) $$\left\{\begin{array}{c} x=r\cos(\Phi )\;\\ y=r\sin(\Phi ). \end{array}\right.$$
  Substituting these into the line equation y=a·x+b, we obtain:
  (31) rsin(Φ)=arcos(Φ)+b
  (32) $$\Rightarrow r=\frac{b}{\sin(\Phi )-a\cos(\Phi )}$$
  which allows us to determine *x* and *y*.
2. Combining the equation y=ax+b with (16), we simplify (16) as follows:
  (33) $$v_{\mathrm{sound}}\tau_{12}=\sqrt{{\left(x-R\right)}^{2}+y^{2}}-\sqrt{{\left(x+R\right)}^{2}+y^{2}}$$
  (34) $$\Rightarrow -\sqrt{{\left(x+R\right)}^{2}+y^{2}}=\left(\frac{2R}{v_{\mathrm{sound}}\tau_{12}}\right)x+\left(\frac{v_{\mathrm{sound}}\tau_{12}}{2}\right).$$
  Substituting y=ax+b into (34) results in:
  (35) $$Ax^{2}+Bx+C=0$$
  where
  (36) $$\left\{\begin{array}{c} A=1+a^{2}-{\left(\frac{2R}{v_{\mathrm{sound}}\tau_{12}}\right)}^{2}\\ B=2ab\\ C=R^{2}+b^{2}-{\left(\frac{v_{\mathrm{sound}}\tau_{12}}{2}\right)}^{2} \end{array}\right.$$
  Solving this equation yields *x*, which can then be substituted into y=ax+b to determine *y*.
3. Combining equation y=ax+b with (17), we first simplify (17) as:
  (37) $$v_{\mathrm{sound}}\tau_{13}=\sqrt{{\left(x-R\right)}^{2}+y^{2}}-\sqrt{x^{2}+{\left(y-R\right)}^{2}}$$
  (38) $$\Rightarrow \sqrt{x^{2}+{\left(y-R\right)}^{2}}=\frac{R}{v_{\mathrm{sound}}\tau_{13}}\left(y-x\right)-\frac{v_{\mathrm{sound}}\tau_{13}}{2}.$$
  Substituting y=ax+b into (37) and (38) results in:
  (39) $$where\;\left\{\begin{array}{c} Ax^{2}+Bx+C=0\;\\ A=1+a^{2}-{\left(\frac{(a-1)R}{v_{\mathrm{sound}}\tau_{13}}\right)}^{2}\;\\ B=2ab-R\left(a+1\right)-2b\left(a-1\right){\left(\frac{R}{v_{\mathrm{sound}}\tau_{13}}\right)}^{2}\;\\ C={\left(b-R\right)}^{2}-\frac{bR}{v_{\mathrm{sound}}\tau_{13}}-{\left(\frac{v_{\mathrm{sound}}\tau_{13}}{2}\right)}^{2} \end{array}\right.$$
  Solving for *x* and substituting into y=ax+b gives *y*.
4. Merging equation y=ax+b with (18), we begin by simplifying (18):
  (40) $$v_{\mathrm{sound}}\tau_{23}=\sqrt{{\left(x+R\right)}^{2}+y^{2}}-\sqrt{x^{2}+{\left(y-R\right)}^{2}}$$
  (41) $$\Rightarrow \sqrt{x^{2}+{\left(y-R\right)}^{2}}=\frac{R}{v_{\mathrm{sound}}\tau_{23}}\left(y+x\right)-\frac{v_{\mathrm{sound}}\tau_{23}}{2}.$$
  Solving this equation provides *x*, which can then be substituted into y=ax+b to obtain *y*.

### 2.5. Extending Localization to Three Dimensions

For the 3D case, we consider a 2D plane that includes the source position and the *x*-axis, forming an angle θ with the *x*-*y* plane. We refer to this as the source-plane (Figure 1). This plane also contains microphones 1 and 2. Under the far-field assumption, microphone 3 can be approximated as lying within this plane with negligible error. Based on these assumptions, and using Equation (29), we can determine θ, which represents the angle of arrival within the source-plane. This value is equivalent to the angle of arrival in the *x*-*y* plane.

Furthermore, using Equation (32), we can calculate *r*, which denotes the distance from the source to the origin. Alternatively, employing Equations (35), (36), or (39), we can derive the coordinates *x* and *y* within the source-plane, rather than the *x*-*y* plane, allowing us to compute *r*. To refine this estimation, we introduce an additional microphone (mic4 in Figure 1) positioned at x=0 and z=y=R, which facilitates the calculation of θ. Under the far-field assumption, microphones 3 and 4 can be approximated as being nearly aligned along the *z*-axis. Consequently, using Equation (29), we obtain θ as

(42) $$\cos\left(\theta \right)=90-\cos^{-1}\left(\sqrt{1-{\left(\frac{v_{\mathrm{sound}}\tau_{34}}{R}\right)}^{2}}\right).$$

With the computed values of *r*, Φ, and θ, the three-dimensional coordinates *x*, *y*, and *z* can be determined as

(43) $$\left\{\begin{array}{c} x=r\cos(\Phi )\sin(\theta )\;\\ y=r\sin(\Phi )\sin(\theta )\;\\ z=r\cos(\theta ). \end{array}\right.$$

This approach enables the estimation of an obstacle’s three-dimensional position using a single microphone array. Since this technique calculates the angle of arrival based on the azimuth angle, it provides accurate localization when the obstacle is at a low elevation. However, at higher elevations, the accuracy of 3D localization declines. To improve localization performance, it is essential to separately estimate the azimuth and elevation angles.

## 3. Methods

### 3.1. Microphone Array Configuration for TDOA Estimation

The position of the sound source is determined by first calculating the TDOA using the spatial separation between microphones and then using the TDOA to estimate the angle information. The spatial separation between microphones allows for the calculation of the TDOA, which provides information on the time delays between received signals. These TDOA values are then used to determine the angle information, providing directional information about where the sound is coming from. Utilizing the microphone array configuration depicted in Figure 1, the angular information of the drone can be extracted, which is crucial for accurately determining its position [12]. Extracting angular information is crucial for accurately determining the drone’s direction and position, which is essential for effective localization. The coordinates of the sound source are represented as $\left(x_{s},y_{s},z_{s}\right)$, while the coordinates of the *n*-th microphone are expressed as $\left(x_{n},y_{n},z_{n}\right)$. The Euclidean distance between these two points is defined by Equation (44).

(44) $$d_{n}=\sqrt{{\left(x_{s}-x_{n}\right)}^{2}+{\left(y_{s}-y_{n}\right)}^{2}+{\left(z_{s}-z_{n}\right)}^{2}}$$

The time at which the sound is received by the *n*-th microphone is denoted as $T_{n}$, while the reception time at the *m*-th microphone is represented as $T_{m}$. The speed of sound, which plays a crucial role in determining the time delay, is approximated as 340 m/s in this study. While Equation (45) provides a more detailed calculation of the speed of sound based on temperature, in this research, a constant value of 340 m/s was used for simplicity. Environmental factors such as humidity and altitude were not explicitly accounted for.

(45) $$V_{sound}=331.3+0.606\times Temperature$$

The time difference of arrival, denoted as $\tau_{nm}$, and the distance difference $d_{nm}$ between the *n*-th and *m*-th microphones are related through the distance–velocity equation and can be expressed as follows:

(46) $$\tau_{nm}=T_{n}-T_{m}=\frac{d_{n}}{V_{sound}}-\frac{d_{m}}{V_{sound}}$$

(47) $$d_{nm}=d_{n}-d_{m}=\tau_{nm}\times V_{sound}$$

The time delay differences between the microphones are represented by Equations (46) and (47) and are computed as detailed in Equations (48)–(51). Each of these equations serves a specific purpose: Equations (46) and (47) represent the initial time delay relationships, while Equations (48)–(51) provide detailed calculations for each microphone pair, allowing for accurate determination of angular and positional information.

(48) $$\tau_{12}=\frac{\sqrt{{\left(x-R\right)}^{2}+y^{2}+z^{2}}}{V_{sound}}-\frac{\sqrt{{\left(x+R\right)}^{2}+y^{2}+z^{2}}}{V_{sound}}$$

(49) $$\tau_{13}=\frac{\sqrt{{\left(x-R\right)}^{2}+y^{2}+z^{2}}}{V_{sound}}-\frac{\sqrt{x^{2}+{\left(y-R\right)}^{2}+z^{2}}}{V_{sound}}$$

(50) $$\tau_{23}=\frac{\sqrt{{\left(x+R\right)}^{2}+y^{2}+z^{2}}}{V_{sound}}-\frac{\sqrt{x^{2}+{\left(y-R\right)}^{2}+z^{2}}}{V_{sound}}$$

(51) $$\tau_{34}=\frac{\sqrt{x^{2}+{\left(y-R\right)}^{2}+z^{2}}}{V_{sound}}-\frac{\sqrt{x^{2}+y^{2}+{\left(z-R\right)}^{2}}}{V_{sound}}$$

### 3.2. Estimation of Angular Information

In this paper, the location of the drone is estimated using angle information, including AOA, azimuth, elevation, and a distributed microphone array. The preliminary estimation of angle information is critical, as it provides the basis for accurately determining the drone’s position. Without an initial estimate of angles such as AOA and elevation, subsequent calculations for precise localization would suffer from reduced accuracy. Elevation and AOA are derived from time difference values, with the required TDOA for each angle calculated using the GCC-PHAT method. The GCC-PHAT method is particularly suitable for this application because it emphasizes phase information, reducing the influence of noise and improving the accuracy of time delay estimation in challenging acoustic environments. Furthermore, the azimuth angle is derived by combining the earlier elevation and AOA. Specifically, the elevation provides information about the vertical orientation, while the AOA helps determine the direction of the sound source relative to the microphone array. By using both, the azimuth angle can be accurately calculated to pinpoint the drone’s location in three-dimensional space.

The arrival angle represents the direction at which the sound source reaches the microphone array, and by accurately estimating this angle, the location of the sound source can be determined with greater precision. In this study, the arrival angle ϕ is estimated based on the TDOA between the microphones. As depicted in Figure 2, with microphones 1 and 2 positioned, the channel environment is assumed to be in the far-field condition, allowing the arrival angle, which is defined as the angle between segment A and the x-axis, to be approximated. The far-field condition is assumed because it simplifies the calculation by allowing the sound waves to be treated as nearly parallel, reducing the complexity of the geometry involved in angle estimation. In this context, the far-field condition assumes that the distance between the sound source and the microphones is sufficiently large, such that the sound waves are nearly parallel when reaching the microphone array, satisfying the following condition.

(52) $$d_{FF}\gg \frac{D_{FF}^{2}}{\lambda }$$

Here, $d_{FF}$ represents the distance between the sound source and the origin of the microphone array, $D_{FF}$ denotes the maximum spacing between the microphones, and λ refers to the wavelength of the signal. The condition for satisfying the far-field approximation is given as follows, which ensures that the sound waves can be considered nearly parallel, thereby simplifying the geometric calculations involved in determining angles.

(53) $$\cos\left(b\right)\approx \frac{C/2}{D/2}$$

(54) C=Dcos(b)

In this context, *b* refers to the angle formed between the y-axis and line A, while *D* represents the separation between microphones 1 and 2. The arrival angle is calculated using Equations (55)–(59).

(55) $${\left(d_{2}-d_{1}\right)}^{2}=D^{2}+C^{2}-2CD\cos\left(b\right)$$

(56) $$b=\cos^{-1}\left(\sqrt{1-{\left(\frac{d_{1}-d_{2}}{D}\right)}^{2}}\right)$$

(57) $$\phi =\frac{\pi }{2}-\cos^{-1}\left(\sqrt{1-{\left(\frac{d_{1}-d_{2}}{D}\right)}^{2}}\right)$$

(58) $$\phi =\frac{\pi }{2}-\cos^{-1}\left(\sqrt{1-{\left(\frac{d_{1}-d_{2}}{2R}\right)}^{2}}\right)$$

(59) $$\phi =\frac{\pi }{2}-\cos^{-1}\left(\sqrt{1-{\left(\frac{v_{sound}\cdot \tau_{12}}{2R}\right)}^{2}}\right)$$

Elevation estimation involves calculating the *z*-axis position of the drone. In a far-field condition, the elevation angle between the origin and the drone is determined using the vertical line *B*, which bisects the distance between microphones 3 and 4. As illustrated in Figure 3, microphone 3 is positioned at (0,R,0) and microphone 4 at (0,R,R), with a vertical separation of *R* between them. With this setup and the time difference of arrival $\tau_{34}$, the elevation angle can be determined. The elevation angle θ is derived as follows:

(60) $$\theta =\frac{\pi }{2}-\cos^{-1}\left(\sqrt{1-{\left(\frac{d_{3}-d_{4}}{R}\right)}^{2}}\right)$$

(61) $$\theta =\frac{\pi }{2}-\cos^{-1}\left(\sqrt{1-{\left(\frac{v_{sound}\cdot \tau_{34}}{R}\right)}^{2}}\right)$$

**Figure 4:**
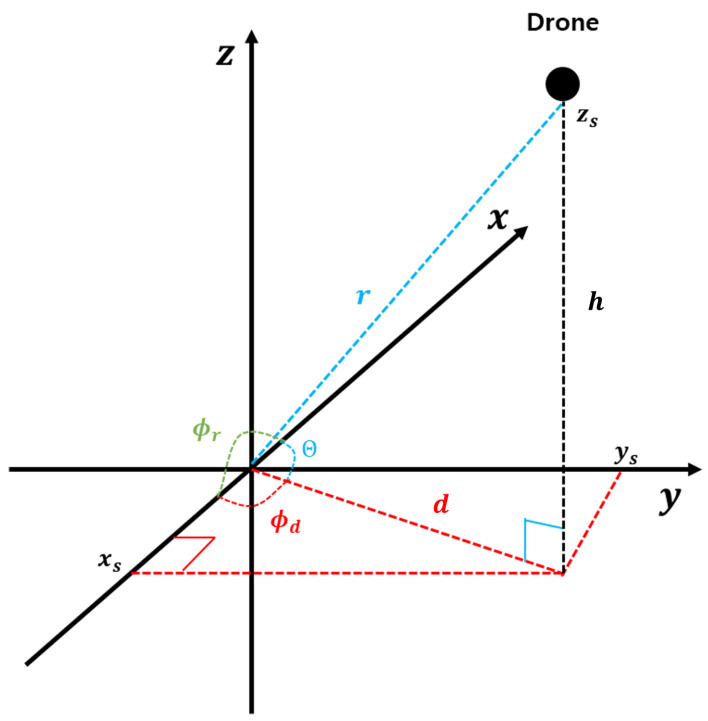
The position of the drone in spherical coordinates. This figure illustrates the drone’s position in a spherical coordinate system, facilitating the understanding of how the AOA, azimuth, and elevation angles are utilized to estimate the drone’s precise coordinates. By analyzing these angles in conjunction, the system can accurately determine the drone’s position in three-dimensional space, improving the overall localization accuracy.

The AOA and elevation angle of the drone were estimated using TDOA. Previous research approximated the AOA ϕ as the azimuth angle; however, ϕ refers to the angle between line A and the x-axis, and this approximation introduces increasing errors as the drone’s z-coordinate rises. Therefore, calculating the azimuth angle accurately becomes crucial. Based on the distance variables presented in Figure 4, the AOA ϕ, azimuth angle $\phi_{d}$, and elevation angle θ are expressed as follows:

(62) $$\phi_{r}=\cos^{-1}\left(\frac{x_{s}}{r}\right)=\cos^{-1}\left(\frac{x_{s}}{\sqrt{d^{2}+h^{2}}}\right)$$

The procedure for deriving the azimuth angle using the equation above is outlined as follows.

(63) $$\phi_{r}=\cos^{-1}\left(\frac{x_{s}}{d}\right)$$

(64) $$\theta =\cos^{-1}\left(\frac{d}{r}\right)$$

(65) $$x_{s}=r\times \cos\left(\phi_{r}\right)$$

By substituting Equations (65) and (66) into Equation (63), Equation (67) is obtained. Equation (67) is significant as it enables the computation of the distance component in the spherical coordinate system, which is essential for accurately calculating the azimuth and elevation angles required for accurate localization.

(66) d=r×cos(θ)

(67) $$\phi_{d}=\cos^{-1}\left(\frac{\cos(\phi_{r})}{\cos(\theta )}\right)$$

As demonstrated above, all angular information in the spherical coordinate system can be computed using TDOA and the microphone array configuration.

### 3.3. Localization Using Distributed Arrangement

**Figure 5:**
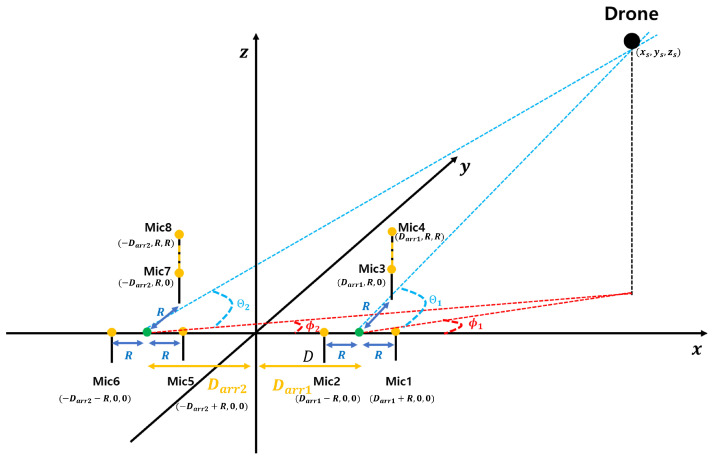
Distributed microphone arrangement. This figure illustrates the distributed placement of the microphone arrays, which improves the localization performance compared to centralized configurations. By strategically dispersing the microphones, the system can capture more spatial information, enabling more accurate estimation of the drone’s three-dimensional coordinates. This configuration is utilized in this study to enhance the precision of the drone’s localization process.

Distributed arrangement is a technique used to more accurately estimate the position of the drone’s sound source. This approach reduces interference between the microphone arrays and provides better differentiation of signal arrival times, leading to improved localization accuracy. In this paper, as shown in Figure 5, two microphone arrays were distributed, and the drone’s coordinates were estimated through the angle information obtained from each array. The following equation expresses $y_{s}$ through the line connecting the reference point of each array and the xy coordinates of the drone.

(68) $$y_{s}=\tan\left(\phi_{1}\right)\left(x_{s}-D_{arr1}\right)$$

(69) $$y_{s}=\tan\left(\phi_{2}\right)\left(x_{s}+D_{arr2}\right)$$

Here, $D_{arr1}$ and $D_{arr2}$ are equal, representing the distance from the origin to the reference points of each array. Using Equations (68) and (69), $x_{s}$ is expressed as shown in Equation (70), where $\phi_{1}$ and $\phi_{2}$ denote the azimuth angles estimated from each microphone array.

(70) $$x_{s}=D_{arr}\times \frac{\tan\left(\phi_{1}\right)+\tan\left(\phi_{2}\right)}{\tan\left(\phi_{1}\right)-\tan\left(\phi_{2}\right)}$$

By applying distributed placement as described above, the intersection of the two lines can be used to estimate $x_{s}$ based on the speed of sound $V_{sound}$ and the TDOA $\tau_{nm}$. This estimated $x_{s}$ is then used to determine $y_{s}$. As illustrated in Figure 5, $z_{s}$ can be derived from the intersection of two lines connecting the origin of each microphone array and the drone, enabling the calculation of the drone’s elevation angle θ. The estimation processes for $z_{s}$ and $y_{s}$ are independent of each other and can be calculated separately for each array. By averaging these estimates, as shown in the following equations, noise robustness is improved [22]. Averaging helps to reduce random errors by smoothing out fluctuations, resulting in a more stable and accurate estimate of the coordinates.

(71) $$\tan\left(\theta_{1}\right)=\frac{z_{s1}}{\sqrt{{\left(x_{s}-D_{arr}\right)}^{2}+y_{s}^{2}}}+n_{1}$$

(72) $$z_{s1}=\left(\tan\left(\theta_{1}\right)-n_{1}\right)\times \sqrt{{\left(x_{s}-D_{arr}\right)}^{2}+y_{s}^{2}}$$

(73) $$\tan\left(\theta_{2}\right)=\frac{z_{s2}}{\sqrt{{\left(x_{s}+D_{arr}\right)}^{2}+y_{s}^{2}}}+n_{2}$$

(74) $$z_{s2}=\left(\tan\left(\theta_{2}\right)-n_{2}\right)\times \sqrt{{\left(x_{s}+D_{arr}\right)}^{2}+y_{s}^{2}}$$

At this stage, $n_{1}$ and $n_{2}$ represent measurement errors, and the estimated $z_{s}$, obtained by averaging the elevation angle estimates, is as follows.

(75) $$z_{s}=\frac{z_{s1}+z_{s2}}{2}$$

Similarly, $y_{s}$ is calculated using the same approach, enabling the determination of the drone’s three-dimensional coordinates.

## 4. Performance Evaluation

Before implementing the actual experiment, simulations were performed to validate the effectiveness of the algorithm. The simulations assessed the impact of noise, evaluated different drone positions, and tested the accuracy of angle estimation. These validations provided insights into the expected performance under varying conditions, helping to ensure the robustness of the approach. In the experiment, the xy coordinates of the drone were arranged according to the predefined experimental setup, and the z-axis coordinates were set to 4 m and 6 m, respectively, to evaluate performance under various conditions.

### 4.1. Simulation Results

In the simulation, a drone positioned 10 m above the origin was placed on a circular path with a radius of 10 m at intervals of approximately 10°, spanning from 0° to 180°. A circular path was chosen to simulate diverse drone positions relative to the microphones, ensuring a comprehensive evaluation of localization accuracy under varying angles. The microphone configuration was identical to that shown in Figure 5, with the distance between the origin of each microphone array and the microphone in the xy-plane set to 1 m and the distance from the origin of the coordinate system to the origin of each microphone array set to 2 m. Additionally, it was assumed that acoustic data were collected under conditions simulating a real channel environment, focusing primarily on noise levels to evaluate localization performance realistically. The focus on noise levels was incorporated to create a realistic scenario for evaluating localization performance. The localization performance was evaluated by calculating the mean error over 1000 trials, defined as the distance between the estimated and actual coordinates. The number of trials was chosen to ensure statistical reliability and robustness of the results, reducing the impact of random variations in the measurements.

The channel environment was assumed to be a Line-of-Sight scenario, accounting for path loss, which was modeled using the Free Space Path Loss formula. This approach allowed for quantifying the reduction in signal strength over distance, providing a realistic evaluation of the channel conditions. To compare the performance of the proposed method with the baseline method, localization accuracy was evaluated in both Additive White Gaussian Noise (AWGN) and ideal channel conditions. These specific channel conditions were chosen to represent both realistic noise scenarios and an optimal environment without interference, providing a balanced comparison that highlights the strengths and limitations of each method under different conditions. The baseline method employed a localization technique that did not consider the azimuth angle, relying solely on elevation angle and TDOA measurements for positioning.

**Figure 6:**
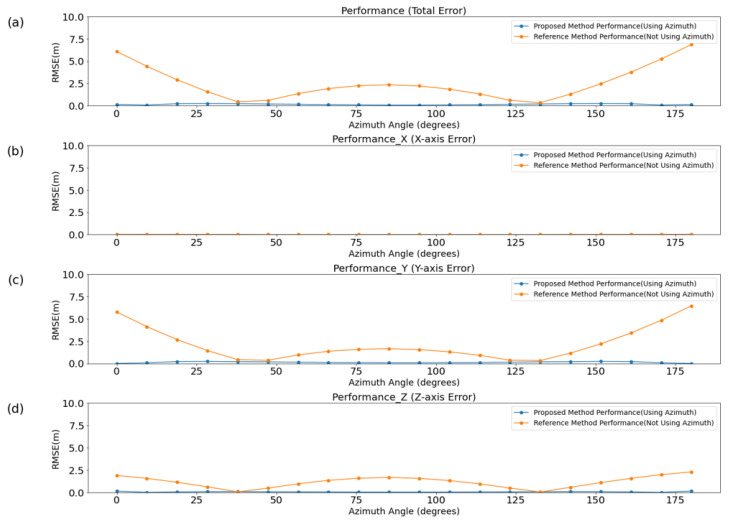
A comparison of localization performance based on the azimuth angle of the drone. This figure analyzes the performance differences between the baseline method and the proposed method under ideal, noise-free conditions to demonstrate the effectiveness of the proposed approach. (**a**) represents the overall localization error, while (**b**) shows the error along the x-axis, (**c**) presents the error along the y-axis, and (**d**) illustrates the error along the z-axis. The results highlight the improved accuracy of the proposed method across all axes in comparison to the baseline.

To evaluate the performance of each method in relation to the azimuth angle, simulations were conducted in an ideal channel, with the results presented in Figure 6. Table 1 and Table 2 display the mean and variance of the overall error, as well as axis-specific errors, for each method. Even in an ideal environment, the difference between the AOA and azimuth, caused by the drone’s altitude, led to an increase in the mean and variance of errors across all axes except the x-axis. The x-axis did not experience an increase in error because the horizontal positioning remained relatively unaffected by changes in altitude, which primarily influenced the y- and z-axes.

**Figure 7:**
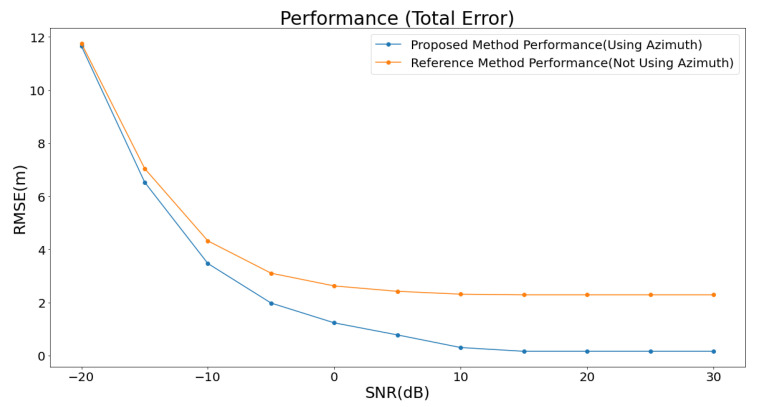
A comparison of localization performance under noisy conditions. This figure illustrates the variation in localization error between the baseline method and the proposed method as a function of the SNR. The graph highlights how the localization accuracy changes under different noise levels, demonstrating the superior robustness of the proposed method in noisy environments compared to the baseline.

Additionally, the performance of each method was analyzed based on the SNR, as illustrated in Figure 7.

The analysis was conducted by incrementally increasing the SNR in steps of 5 dB, ranging from −20 dB to 30 dB. For the proposed method, the average error decreased to under 3 m when the SNR exceeded 0 dB. It further dropped to below 0.3 m when the SNR surpassed 15 dB. At SNR levels above 20 dB, the average error converged to approximately 0.16 m. In contrast, the baseline method showed a tendency to stabilize at an average error of about 2.5 m, even when the SNR increased beyond 15 dB. When the SNR was below 0 dB, the performance difference between the two methods was less than 1 m. However, as the SNR exceeded 0 dB, a significant performance gap emerged, ranging from 1 m to a maximum of 2.34 m. This performance difference can be attributed to the proposed method’s effective utilization of angular information, which becomes increasingly beneficial as the signal quality improves with higher SNR.

### 4.2. Field Test

In this study, field tests were conducted with drones positioned at various locations to evaluate the performance of the proposed algorithm across different coordinates and verify its effectiveness in real-world conditions.

**Figure 8:**
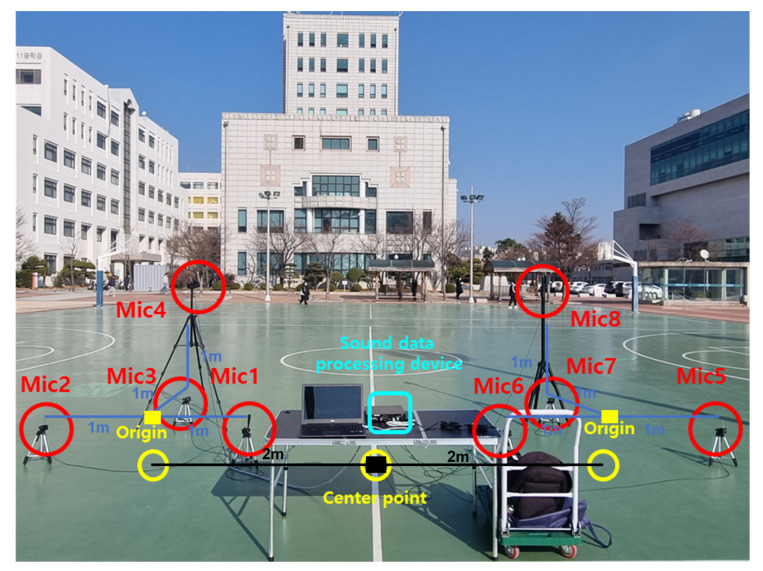
Microphone deployment setup for field testing. This figure depicts the distributed microphone arrangement utilized during field testing. The setup was designed to optimize the capture of acoustic signals in a real-world environment, facilitating accurate localization of the drone’s three-dimensional coordinates. The strategic placement of the microphones ensures robust data collection, even in complex and noisy conditions, and enhances the overall effectiveness of the localization algorithm.

The acoustic signals generated by the drones were captured using an array of eight PCB Piezotronics 130F22 microphones (PCB Piezotronics, Depew, NY, USA), each with a 0.25-inch diaphragm, 45 mV/Pa sensitivity, and a frequency response of 10 Hz–20 kHz. These microphones were configured in a distributed array, as shown in Figure 8, to enhance localization accuracy by leveraging the time difference of arrival and the GCC-PHAT techniques.

The microphone arrays were deployed in a structured configuration, ensuring optimal coverage for wide-area drone localization. The microphone configuration was identical to that shown in Figure 8, with the distance between the center of each microphone array and the microphones in the xy-plane set to 1 m, while the distance from the coordinate system’s origin to the center of each microphone array was 2 m. This arrangement was designed to maximize localization performance while maintaining cost efficiency in large-scale deployments.

Each microphone was connected to a high-speed USB audio interface, and the signals were processed in real time by the NVIDIA Jetson AGX Xavier (NVIDIA Corporation, Santa Clara, CA, USA), which features an 8-core ARM CPU and a 512-core NVIDIA Volta GPU optimized for signal processing. The Jetson system performed real-time data acquisition and localization calculations, integrating the GCC-PHAT algorithm to estimate the drone’s position with high precision.

**Figure 9:**
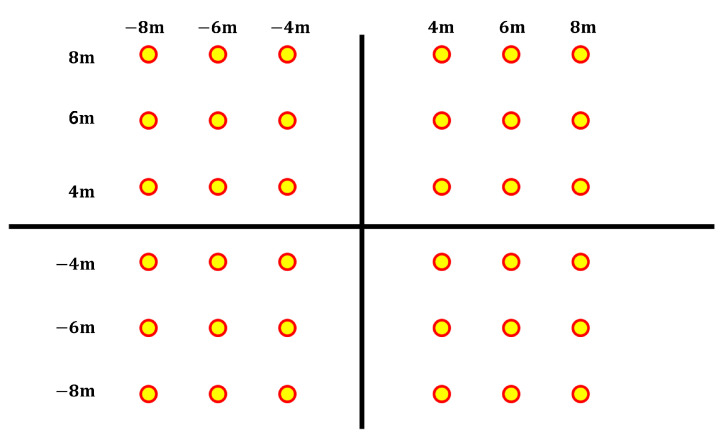
A drone deployment diagram of the experimental setup. This figure illustrates the coordinates of the drone deployment used during the experiment. Although the Z-axis coordinates are not shown in the figure, they were set to a fixed value of 5 m for the duration of the experiment.

The localization error was defined as the distance between the actual drone coordinates and the estimated positions. The drones were positioned at the xy coordinates shown in Figure 9, with altitudes of 4 m and 6 m, respectively. These altitudes were slightly reduced to ensure accurate evaluation, as the low signal strength of the drones used in the experiment posed measurement challenges.

Due to the low acoustic intensity of the drone’s emitted sound, the maximum experimental distance was limited to 10 m to ensure reliable detection. Despite this constraint, the proposed localization method demonstrated accurate performance within this range, validating its feasibility.

Furthermore, the planar wave approximation remains applicable in this setup, as the wavelength of the drone’s sound is relatively short compared to the microphone array’s spatial configuration, minimizing spherical wavefront distortions.

Following the placement of the drones, 10 measurements were taken at each location, and the average values were used to evaluate the localization performance. The results, as shown in Table 3 and Table 4, demonstrate the performance of the proposed algorithm in comparison to the baseline method.

As can be seen from the tables, the proposed method showed an average error reduction of approximately 1.7 m in all configurations of the real-world test environment. The variance was also reduced by approximately 0.9575 m^2^. These reductions demonstrate consistent localization performance across various angles, indicating the method’s effectiveness in minimizing errors even under different conditions.

While the baseline method outperformed its simulation results in real-world tests, the proposed method demonstrated slightly reduced performance under real-world conditions. This discrepancy can be explained by two key factors. First, significant background noise during the experiments led to a lower SNR, which negatively impacted the performance of the proposed method. Second, in the experimental setup, the drone was positioned 5 m lower than in the simulations due to difficulties in collecting sound signals at altitudes above 10 m. This adjustment reduced the angular difference between the azimuth and AOA. This reduction in angular disparity contributed to the improved performance of the baseline method in comparison to the simulation results.

**Figure 10:**
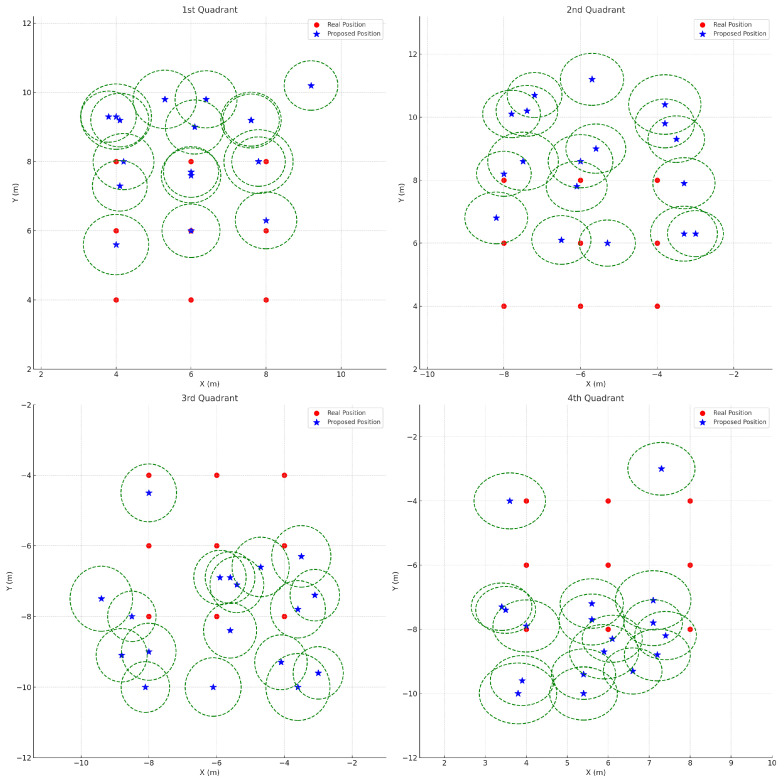
Comparison method localization results across four quadrants. Red circles represent the drone’s real position, while blue stars denote the estimated positions obtained via the comparison method. The green dashed circles depict the 95% confidence intervals, computed using the average standard deviations across all quadrants: $\sigma_{x}=0.1302$, $\sigma_{y}=1.1345$, $\sigma_{z}=0.3556$, a sample size of n=10, and $t_{\alpha /2}=2.26$. These values represent the mean standard deviation of localization errors across all measured positions. Overall, this comparison method exhibits relatively large localization errors in each quadrant—particularly noticeable in the 2nd and 4th quadrants—indicating limited performance for sound sources at higher altitudes.

In Figure 10, the error bars—depicted as green dashed circles—illustrate the 95% confidence intervals of the localization results obtained using the comparison method. These intervals were computed using the average standard deviations across all quadrants ($\sigma_{x}=0.1302$, $\sigma_{y}=1.1345$, $\sigma_{z}=0.3556$), a sample size of n=10, and a *t*-value of 2.26. Notably, the comparison method exhibits relatively large localization errors in each quadrant, particularly along the y-axis in the second and fourth quadrants. This suggests that the baseline approach, which does not adequately account for the discrepancy between the azimuth and the angle of arrival (AOA), struggles to accurately estimate the true direction of the sound source, especially when the source is at a significant altitude. The resulting reliance solely on the AOA leads to increased errors, as evidenced by the wide error bars.

Moreover, because the estimation of the z-axis coordinate depends on the accuracy of the x- and y-axis estimates, the large variance in y-axis localization further degrades the performance of z-axis estimation. The overall large error bars in Figure 10 serve as a clear indication of the increased uncertainty associated with this method.

**Figure 11:**
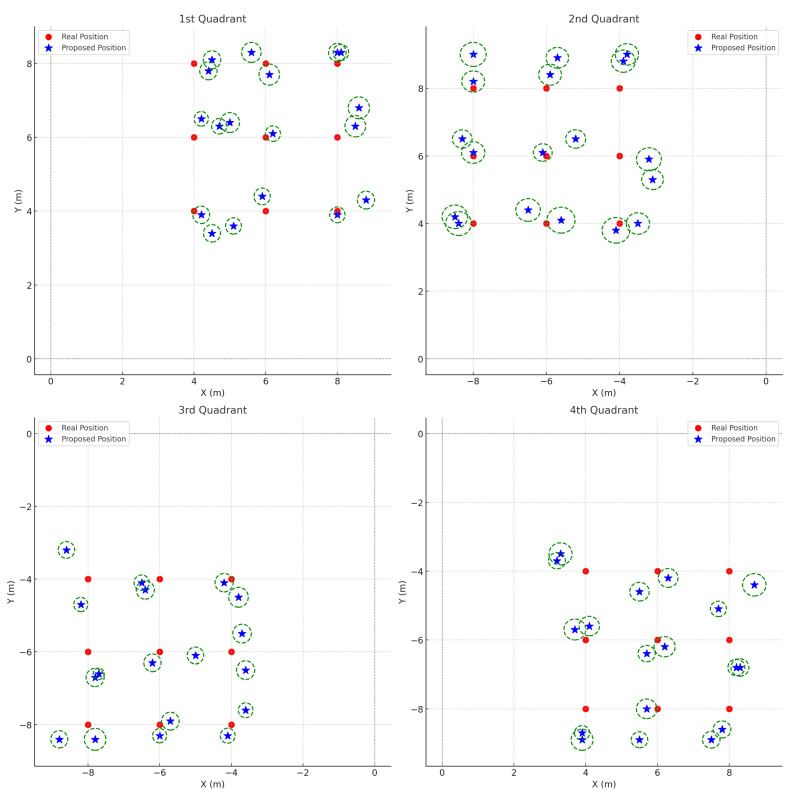
Proposed method localization results across four quadrants. Red circles represent the drone’s real position, while blue stars denote the estimated positions obtained via the comparison method. The green dashed circles depict the 95% confidence intervals, computed using the average standard deviations across all quadrants: $\sigma_{x}=0.2854$, $\sigma_{y}=0.1934$, $\sigma_{z}=0.2081$, a sample size of n=10, and $t_{\alpha /2}=2.26$. These values represent the mean standard deviation of localization errors across all measured positions.

In contrast, Figure 11 shows the localization results achieved using the proposed method. Although the same confidence interval calculation approach is used, the average standard deviations here ($\sigma_{x}=0.2854$, $\sigma_{y}=0.1934$, $\sigma_{z}=0.2081$) differ, reflecting a refined estimation process. Consequently, the proposed method demonstrates reduced localization errors and lower variance—particularly along the y- and z-axes—as compared to the baseline method. The smaller error bars in Figure 11 indicate that incorporating azimuth angle calculations into the algorithm substantially improves localization accuracy for sound sources at high altitudes.

Overall, the comparison between Figure 10 and Figure 11 demonstrates that the baseline (comparison) method suffers from significant localization uncertainty, whereas the proposed method offers superior performance by effectively integrating azimuth information. This enhancement is critical for applications such as drone detection, where precise 3D localization is essential.

## 5. Discussion

This paper proposed a localization technique that employs distributed placement and azimuth angle estimation to accurately determine the position of high-altitude drones. The effectiveness of the proposed method was validated through both simulations and real-world experiments.

The simulation results demonstrated that the localization error of the proposed method was significantly lower than that of the baseline method in an ideal channel. Additionally, the proposed method outperformed the baseline in noisy environments with SNRs ranging from −20 dB to 30 dB, with the performance gap widening as the SNR increased. Real-world test results further confirmed the superior localization accuracy of the proposed method across various locations. On average, the proposed method achieved a 1.7 m reduction in error compared to the baseline method, along with a variance reduction of 0.9575 m^2^. These findings indicate that incorporating azimuth angle estimation enhances both the accuracy and precision of drone localization systems.

While the proposed method has demonstrated significant improvements in drone localization accuracy, several areas remain for future exploration and enhancement.

One critical direction for future research is the extension of the proposed method to multiple drone scenarios (Drone Swarm Localization). The current approach focuses on localizing a single drone at a time; however, real-world applications often involve detecting and tracking multiple drones simultaneously. To address this, future studies could explore multi-source localization techniques, such as array signal processing for simultaneous TDOA estimation or machine learning-based source separation methods.

Another important research direction is expanding the operational range of the system. The experimental range was limited to 10 m due to the low acoustic intensity of the drone’s emitted sound, but real-world drone detection scenarios often require localization at distances exceeding 100 m. To extend this capability, future research could integrate high-sensitivity microphones, directional microphone arrays, or advanced signal processing techniques to improve detection at longer distances.

Furthermore, the proposed localization system could be integrated with emerging technologies such as 5G networks and edge computing to enable real-time, distributed processing. Leveraging 5G-enabled IoT infrastructure would allow for low-latency processing and network-based localization, making the system more scalable and efficient for real-world deployment.

Additionally, future research could explore the applicability of AI-driven localization models, which could dynamically adapt to environmental noise conditions and optimize localization performance in challenging urban or battlefield environments. Techniques such as deep learning-based denoising and adaptive filtering could further enhance the robustness of drone acoustic localization.

Lastly, this study focused on passive acoustic localization, but future research could investigate the integration of multi-modal sensor fusion, combining acoustic localization with RF-based positioning, computer vision, or LiDAR-based tracking to achieve higher reliability and accuracy in complex operational environments.

By addressing these challenges and integrating with next-generation technologies, the proposed method has the potential to significantly advance the capabilities of drone localization and surveillance systems for applications in aerospace security, smart cities, and autonomous monitoring systems.

## 6. Conclusions

This study introduced an innovative localization strategy that employs two sets of distributed microphone arrays in combination with the Generalized Cross-Correlation Phase Transform (GCC-PHAT) method to enhance anti-drone detection capabilities. Unlike traditional localization approaches, the proposed method achieves higher precision by accurately estimating the azimuth angle while leveraging the unique acoustic characteristics of drones. The key contribution of this study lies in the development of a robust localization framework that improves both accuracy and stability, even in the presence of noise and environmental uncertainties.

The effectiveness of the proposed system was validated through both simulation and field experiments, demonstrating substantial improvements in localization accuracy over existing techniques. In simulation environments, the proposed method significantly reduced both the mean and variance of localization errors compared to conventional approaches, leading to more precise positioning results. Additionally, in noisy conditions, the proposed system consistently outperformed baseline methods across a wide range of Signal-to-Noise Ratio (SNR) levels, achieving a peak localization improvement of 2.13 m when the SNR exceeded 0 dB. Notably, while traditional methods exhibited decreased accuracy along the y-axis and z-axis, the proposed approach maintained stable performance across all axes, highlighting its robustness in dynamic environments.

Field tests further reinforced the practical applicability and reliability of the proposed localization framework, as real-world experiments produced results highly consistent with simulation findings. By effectively differentiating between azimuth and elevation angles, this study demonstrates that the proposed method provides significant enhancements in localization accuracy, particularly in challenging and dynamic conditions where multi-path interference and environmental noise are prevalent.

In future work, the adaptability of the proposed method will be assessed across various drone types and broader environmental settings. Further comparisons with alternative localization techniques will be conducted to refine the algorithm and enhance real-time performance. Additionally, future research will explore the integration of advanced AI-driven models and 5G-enabled IoT networks to develop a scalable and efficient localization system for real-world deployment.

By addressing these challenges, this study contributes to advancing the field of acoustic drone localization and provides a foundation for future developments in aerospace security, smart surveillance, and autonomous monitoring systems.

## Figures and Tables

**Table 1:** Localization performance by azimuth in the baseline method. This table presents the statistical localization performance of the baseline method, derived from simulation results. It displays the overall mean and variance of the localization error, as well as the mean and variance of the error along each axis (x, y, z). These statistics provide insight into the accuracy and precision of the baseline method for azimuth-based localization.

Axis	Mean (m)	Variance (m^2^)	95% CI (m)
Total Error	2.4906	3.3434	[1.3567, 3.6244]
X-axis Error	0.0108	0.0001	[0.0046, 0.0170]
Y-axis Error	2.1477	3.2090	[1.0368, 3.2586]
Z-axis Error	1.1592	0.3813	[0.7763, 1.5421]

**Table 2:** Localization performance by azimuth in the proposed method. This table presents the statistical localization performance of the proposed method, based on simulation results. It provides the overall mean and variance of the localization error, along with the mean and variance of the error for each individual axis (x, y, z). These metrics demonstrate the accuracy and precision improvements achieved by the proposed method in azimuth-based localization compared to the baseline method.

Axis	Mean (m)	Variance (m^2^)	95% CI (m)
Total Error	0.1569	0.0035	[0.1202, 0.1936]
X-axis Error	0.0108	0.0001	[0.0046, 0.0170]
Y-axis Error	0.1339	0.0052	[0.0892, 0.1786]
Z-axis Error	0.0594	0.0013	[0.0370, 0.0818]

**Table 3:** Experimental statistics of localization performance for the baseline method. This table presents the statistical results of localization performance obtained through experimental testing of the baseline method. The data include the overall mean and variance of the localization error, as well as the error metrics for each axis (x, y, z), providing insight into the method’s accuracy in real-world conditions.

Axis	Mean (m)	Variance (m^2^)	95% CI (m)
Total Error	2.4526	1.0463	[1.8182, 3.0870]
X-axis Error	0.418	0.1302	[0.1942, 0.6418]
Y-axis Error	2.1931	1.1345	[1.5325, 2.8537]
Z-axis Error	0.6819	0.3556	[0.3120, 1.0518]

**Table 4:** Localization performance by azimuth in the proposed method. This table presents the localization performance of the proposed method as a function of the azimuth angle. It includes the overall mean and variance of localization errors, as well as detailed error metrics for each axis (x, y, z). The results demonstrate the effectiveness of the proposed method in accurately estimating the drone’s position based on azimuth data.

Axis	Mean (m)	Variance (m^2^)	95% CI (m)
Total Error	0.7668	0.0888	[0.5820, 0.9516]
X-axis Error	0.3458	0.0653	[0.1873, 0.5043]
Y-axis Error	0.4194	0.0782	[0.2460, 0.5928]
Z-axis Error	0.4125	0.0693	[0.2492, 0.5758]

## Data Availability

The datasets generated and analyzed during this study are partially available on GitHub at the following link, due to security constraints: https://github.com/LimJJ23/Drone_Localization (accessed on 20 December 2024). For access to the full dataset, please contact the authors via email.
